# Proteolytic Activities and Immunological Effects of Light Chains of Botulinum Neurotoxin A1, A2 and A3 Subtypes

**DOI:** 10.3390/toxins18010016

**Published:** 2025-12-26

**Authors:** Yiying Liao, Xin Hu, Jingrong Wang, Jiansheng Lu, Shuo Yu, Yunzhou Yu, Wenhui Wu

**Affiliations:** 1 College of Food Science and Technology, Shanghai Ocean University, Shanghai 201306, China; lyying1128@163.com; 2 Academy of Military Medical Sciences, Beijing 100850, China; huxin1407@163.com (X.H.); wjr010108@163.com (J.W.); lujiansheng2008@163.com (J.L.); o_yys@163.com (S.Y.)

**Keywords:** botulinum neurotoxin, light chain, proteolytic activity, SNAP-25, protective immunity

## Abstract

Botulinum neurotoxin serotype A (BoNT/A) is the most potent known neurotoxin. While its light chain (LC) catalytic domain is a prime target for next-generation vaccines and therapeutics, the functional differences among BoNT/A subtype LCs (A1, A2, A3) remain to be definitively characterized, despite notable sequence variation. This work aimed to systematically compare the proteolytic activity and immunoprotective efficacy of recombinant BoNT/A1-LC, A2-LC, and A3-LC. Recombinant A1-LC-His, A2-LC-His, A3-LC-His, and A3-LC-Twin-Strep proteins were expressed in *Escherichia coli* (*E. coli*) and purified with affinity chromatography. Their proteolytic activity was assessed via in vitro SNAP-25 cleavage assays. The protective potency of these antigens was evaluated in a mouse model. In vitro cleavage assays revealed a substrate cleavage efficiency order of A2-LC > A1-LC > A3-LC. In vivo, both A1-LC and A2-LC immunization conferred robust, broad protection against high-dose challenges with all three toxin subtypes. In stark contrast, A3-LC provided only minimal protection against its homologous toxin and none against heterologous subtypes. Crucially, the functional deficit of A3-LC was confirmed to be an intrinsic property, as the A3-LC-TS variant, designed to exclude tag-specific interference, exhibited comparable low efficacy. According to structural research, A3-LC’s compromised function may be caused by a four-amino-acid loss. The inferior performance of A3-LC is inherent to its primary structure. This work identified A1-LC or A2-LC as the potential proteolytic activity molecule and vaccine antigen by demonstrating functional differences among BoNT/A subtype LCs. These findings provide crucial insights for developing subtype-specific countermeasures against botulism.

## 1. Introduction

Botulinum neurotoxin (BoNT), a protein neurotoxin secreted by *Clostridium botulinum* under anaerobic conditions, is one of the most potent lethal biological toxins known to date [1,2]. It exhibits extreme toxicity, with a median lethal dose (LD_50_) of 1 ng per kg of body weight and can cause foodborne poisoning through contaminated products [3,4]. Based on antigenic specificity, BoNTs are categorized into seven serotypes (A–G). Among them, serotypes A, B, E, and F are associated with human botulism, while types C and D primarily affect animals. Notably, BoNT/A is recognized as causing the most severe and longest-lasting disease [2].

Structurally, BoNT is a ~150 kDa di-chain toxin composed of a ∼100 kDa heavy chain (HC) and a ~50 kDa light chain (LC), linked by a disulfide bond [5]. The HC can be further divided into two functional domains of approximately 50 kDa each: the receptor-binding domain (Hc) and the transmembrane translocation domain (HN) [6]. The Hc domain mediates binding to specific receptors on neuronal surfaces, facilitating toxin internalization. Under the acidic conditions of the endosome, the HN domain undergoes conformational changes to form a transmembrane channel that enables the translocation of the LC into the cytosol. The LC functions as a zinc-dependent protease that cleaves SNARE proteins, which are key components of the neurotransmitter release machinery, thereby blocking synaptic transmission and leading to flaccid paralysis or death [7,8,9].

BoNT/A can be subdivided into eight subtypes (A1–A8) [7,10]. Although these subtypes share overall structural and functional similarities, they also exhibit significant variations that may influence their biological activity and immunogenic properties [11,12]. BoNT/A1 is the most extensively explored and widely applied serotype [13]. However, the characteristics of its light chain (LC) have not been systematically compared with those of other subtypes, such as BoNT/A2 and BoNT/A3. In particular, the functional differences among LCs and their underlying molecular basis remain largely unknown.

Previous studies have indicated notable variations in overall toxicity among BoNT/A1, A2, and A3: native BoNT/A1 is highly potent, BoNT/A2 exhibits comparable or slightly higher toxicity than BoNT/A1, whereas BoNT/A3 is significantly less toxic [12,14]. Most existing antitoxins and vaccines primarily target the full-length toxin or the heavy chain [15]. As the LC serves as the zinc-dependent endopeptidase critical for the lethal neurotoxicity of BoNT, inhibiting its activity represents a fundamental strategy to block toxin function. Nevertheless, vaccines specifically designed against the LC remain limited.

Given the extreme toxicity of native toxins, which are classified as high-risk biological agents, recombinant proteins derived from toxins were expressed in a prokaryotic system [16,17,18]. Recombinant LCs expressed in prokaryotic systems lack the heavy chain domains responsible for neuronal binding and internalization, rendering them inherently non-cytotoxic in animal or human and they are safe during the experiment [16,19,20]. Moreover, this approach ensures high yield and purity [21]. Therefore, this study employed recombinant protein expression and purification instead of isolating LCs from native toxins.

This work focuses on the functional characterization of BoNT/A1, A2, and A3 LCs. Four recombinant proteins—BoNT/A1-LC, BoNT/A2-LC, BoNT/A3-LC-His, and BoNT/A3-LC-TS—were developed in *E. coli* using the pTIG-Trx expression vector and subsequently purified [22]. Following validation, their differences were systematically compared through in vitro proteolytic activity and in vivo protective efficacy experiments in mice. Our work clearly delineates systematic differences among these highly homologous LCs, providing critical insights for understanding BoNT function and mechanism, and facilitating the development of next-generation, subtype-specific antitoxins and therapeutics.

## 2. Results

### 2.1. Protein Purification and Identification

**Figure 1:**
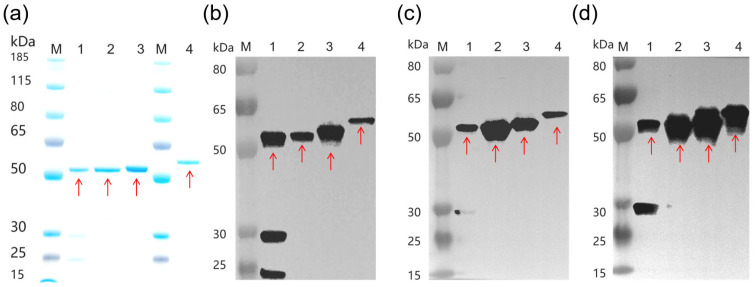
Development and identification of A1-LC-His, A2-LC-His, A3-LC-His and A3-LC-TS proteins. The red arrows indicate the A-LC protein (1 μg/lane). Lane 1: A1-LC-His; Lane 2: A2-LC-His; Lane 3: A3-LC-His; Lane 4: A3-LC-TS. (**a**) SDS-PAGE of A1-LC-His, A2-LC-His, A3-LC-His and A3-LC-TS proteins. (**b**–**d**) Western blotting of A1-LC-His, A2-LC-His, A3-LC-His and A3-LC-TS proteins. Recombinant proteins (0.3 μg/lane) were detected with specific anti-A1-LC (**b**), anti-A2-LC (**c**), anti-A3-LC (**d**) polyclonal antisera, respectively.

Recombinant A1-LC-His, A2-LC-His, A3-LC-His, and A3-LC-TS proteins were successfully expressed in *E. coli* and purified to high homogeneity using their respective affinity tags, as confirmed by SDS-PAGE analysis (Figure 1a). Western blot analysis demonstrated strong antigenic specificity of all four purified recombinant proteins against their corresponding polyclonal antisera (Figure 1b–d). A control blot probed with naïve serum yielded no detectable signal, verifying that the observed immunoreactivities were specific. Furthermore, the three subtype light chain molecules had strong cross-antigenicity between each other and also had different cross-reactivity characteristics with subtype antibodies.

### 2.2. Proteolytic Activity

**Figure 2:**
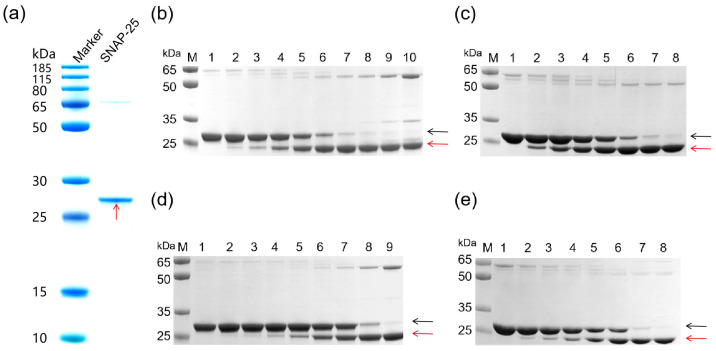
(**a**) SDS-PAGE of SNAP-25 proteins. (**b**–**d**) Electrophoretic analysis of SNAP-25 substrate cleavage by A1-LC, A2-LC, and A3-LC proteins. Black arrows indicate uncleaved SNAP-25, red arrows indicate cleaved SNAP-25. The substrate was fixed at 2.4 μM SNAP-25 in all reactions. Lanes 1, 2.4 μM SNAP-25. (**b**) Cleavage of SNAP-25 by A1-LC-His. Lanes 2–10: 4.7, 9.4, 18.75, 37.5, 75, 150, 300, 600, and 1200 nM A1-LC-His. (**c**) Cleavage of SNAP-25 by A2-LC-His. Lanes 2–8: 1.2, 2.4, 4.7, 9.4, 18.75, 37.5, and 75 nM A2-LC-His. (**d**) Cleavage of SNAP-25 by A3-LC-His. Lanes 2–9: 9.4, 18.75, 37.5, 75, 150, 300, 600, 1200 nM A3-LC-His. (**e**) Cleavage of SNAP-25 by A3-LC-TS. Lanes 2–8: 18.75, 37.5, 75, 150, 300, 600, and 1200 nM A3-LC-TS.

**Figure 3:**
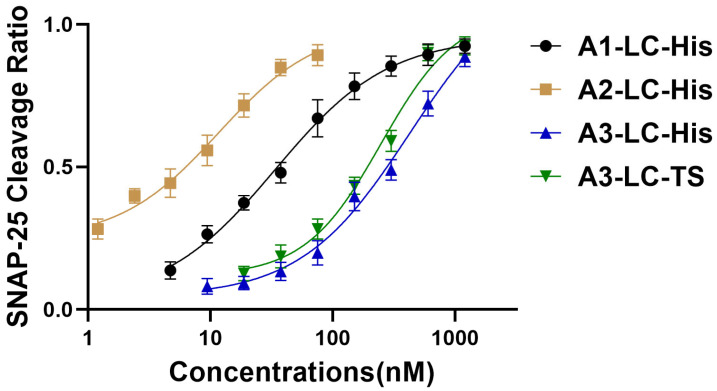
SNAP-25 Cleavage Assay. The value of SNAP-25 cleavage ratio was calculated as the fraction of cleaved SNAP-25 relative to the total SNAP-25 signal (Lane 1, 2.4 μM SNAP-25 from Figure 2). Recombinant proteins A1-LC-His, A2-LC-His, A3-LC-His, and A3-LC-TS at various concentrations were incubated with 2.4 μM SNAP-25 at 37 °C for 30 min. The half-maximal effective concentration (EC_50_) is defined as the toxin concentration required to achieve 50% substrate cleavage.

To validate the proteolytic activity of the recombinant A1-LC, A2-LC, and A3-LC proteins, an in vitro cleavage assay was performed using their substrate, SNAP-25 (Figure 2a). With the SNAP-25 concentration fixed at 2.4 μM and the recombinant proteins serially diluted (resulting in molar ratios of SNAP-25 to recombinant protein decreasing from 2:1 to 1:2048), a concentration-dependent cleavage was observed. The assay for each LC proteolytic activity was independently repeated three times. The results are similar among the three times; only minor variations in band intensity were observed across replicates. Each LC protein consistently exhibited a clear concentration-dependent cleavage trend. Figure 2b–e presents a representative set of results from these replicates. As shown, the intact SNAP-25 band gradually diminished with increasing recombinant protein concentration, accompanied by a corresponding increase in cleavage product bands. Grayscale analysis of the intact substrate bands in Figure 2b–e using ImageJ 1.54g software (NIH, USA) [23] enabled the plotting of recombinant protein concentration versus substrate cleavage percentage (Figure 3). The EC_50_ values derived from this representative experiment indicated that, with the substrate concentration fixed at 2.4 μM, the EC_50_ values for A1-LC-His, A2-LC-His, A3-LC-His, and A3-LC-TS were determined to be 33.93 nM, 11.62 nM, 425 nM, and 247.5 nM, respectively.

### 2.3. Immunization and Protective Efficacy

To evaluate the immunoprotective efficacy of A1-LC-His, A2-LC-His, and A3-LC-His antigens in vivo, Balb/c mice subjected to three or four immunizations were challenged with BoNT/A toxin. Mice were immunized intramuscularly with 1 or 10 μg of antigen formulated with 10% aluminum adjuvant in a total volume of 100 μL at two-week intervals for three or four cycles. Three weeks after the final immunization, mice were challenged intraperitoneally with the corresponding homologous BoNT/A toxin: the A1-LC-His immunization group with BoNT/A1, the A2-LC-His immunization group with BoNT/A2, and the A3-LC-His immunization group with BoNT/A3. Survival was monitored for 7 days post-challenge. Subsequently, a cross-challenge with heterologous toxins was conducted on surviving mice.

The results demonstrated that the 10 μg A1-LC-His immunization group with three immunizations conferred complete protection against challenge with 10 LD_50_ of BoNT/A1 and 10 LD_50_ of BoNT/A3, and an 80% protection rate against 10 LD_50_ of BoNT/A2 (Table 1). The 10 μg A2-LC-His immunization group provided complete protection against 10 LD_50_ of BoNT/A2, BoNT/A1 and BoNT/A3. In contrast, the 10 μg A3-LC-His or A3-LC-TS immunization group failed to protect against challenge with even 10 LD_50_ of its homologous BoNT/A3 toxin.

Following four immunizations against a high-dose challenge of 100 LD_50_ BoNT/A toxin (Table 2), the A2-LC-His immunized group exhibited the most robust protection. The A1-LC-His immunized group provided effective protection. In Table 1 and Table 2, statistical analysis indicated no significant difference in the protective efficacy between the A1-LC and A2-LC groups, suggesting in vivo immunoprotective potencies were comparable under these experimental conditions. The A3-LC-His immunized group offered only weak protection against 100 LD_50_ of BoNT/A3 and failed to protect against 100 LD_50_ of either BoNT/A1 or BoNT/A2. The A3-LC-TS variant after our immunizations consistently showed no significant protective effect. This further confirms that the A3-LC molecule itself possesses weak immunogenic potency.

**Figure 4:**
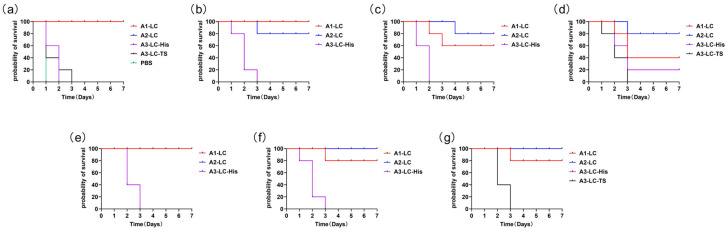
Kaplan–Meier survival analysis of immunized mice challenged with BoNT/A subtypes. (**a**) After three immunizations of 10 μg, challenge with 10 LD_50_ BoNT/A3. (**b**–**d**) after four immunizations of 1 μg, challenge with 100 LD_50_ of BoNT/A1 (**b**), BoNT/A2 (**c**), or BoNT/A3 (**d**). (**e**–**g**) after four immunizations of 10 μg, challenge with 100 LD_50_ of BoNT/A1 (**e**), BoNT/A2 (**f**), or BoNT/A3 (**g**).

As shown in the Kaplan–Meier survival curves (Figure 4), animals in the A1-LC and A2-LC groups that succumbed appeared to die slightly later than those in the A3-LC group. This indicated that immune sera from the former two groups possessed some neutralizing capacity against high-dose challenge, thereby potentially delaying death. Overall, our findings demonstrate that there are protective epitopes intrinsic in LC of BoNT/A, particularly in A2-LC or A1-LC.

### 2.4. Immunogenicity of A1-LC-His, A2-LC-His, A3-LC-His Antigens

**Figure 5:**
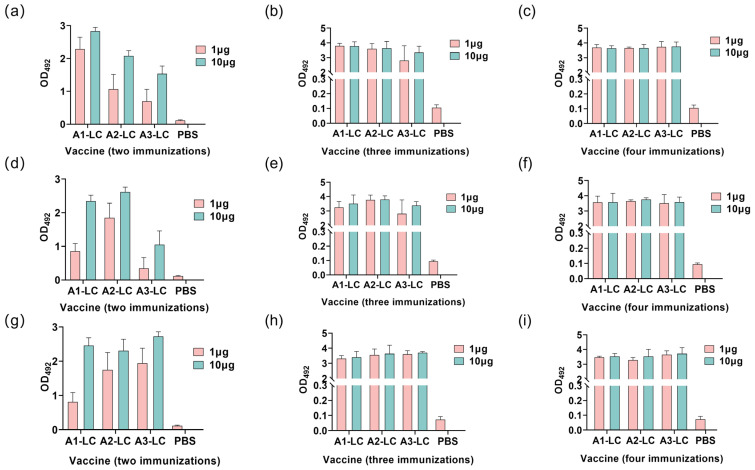
Serum IgG antibody levels in mice immunized with 1 μg or 10 μg of A1-LC, A2-LC, or A3-LC antigens following the second, third, and fourth immunization. (**a**–**c**) Anti-A1-LC antibody responses of A1-LC, A2-LC, or A3-LC antigens after the second immunization (**a**), after the third immunization (**b**), and after the fourth immunization (**c**). (**d**–**f**) Anti-A2-LC antibody responses of A1-LC, A2-LC, or A3-LC antigens after the second immunization (**d**), after the third immunization (**e**), and after the fourth immunization (**f**). (**g**–**i**) Anti-A3-LC antibody responses of A1-LC, A2-LC, or A3-LC antigens after the second immunization (**g**), after the third immunization (**h**), after the fourth immunization (**i**). *n* = 5/group. Data are presented as mean ± SD.

Antigen-specific antibody levels and antibody titers in serum samples from different immunization groups were determined by enzyme-linked immunosorbent assay (ELISA). As shown in Figure 5, a continuous and significant increase in specific IgG antibody levels was observed with successive immunizations. Following the second immunization, relatively low-level antibodies were induced in low-dose immunization groups. A sharp increase in antibody levels was evident after both the third and fourth immunizations in both high- and low-dose groups.

**Figure 6:**
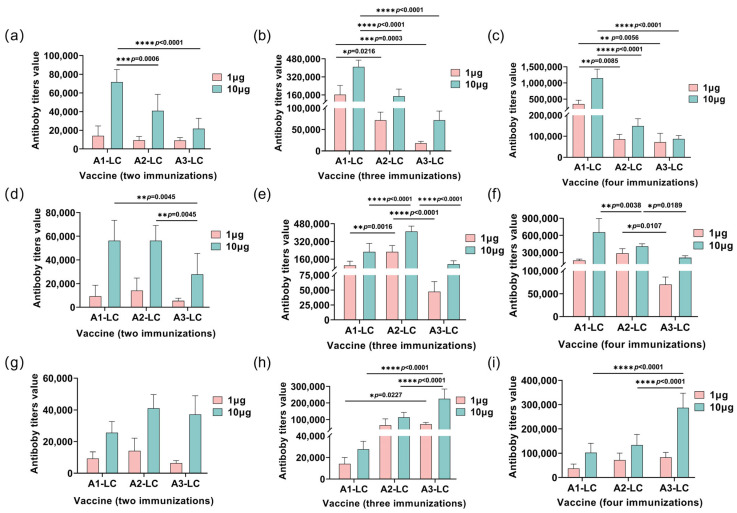
Serum antibody titers in mice immunized with 1 μg or 10 μg of A1-LC, A2-LC, or A3-LC antigens following the second, third, and fourth immunization. (**a**–**c**) Anti-A1-LC antibody titers of A1-LC, A2-LC, or A3-LC antigens after the second immunization (**a**), after the third immunization (**b**), after the fourth immunization (**c**). (**d**–**f**) Anti-A2-LC antibody titers of A1-LC, A2-LC, or A3-LC antigens after the second immunization (**d**), after the third immunization (**e**), after the fourth immunization (**f**). (**g**–**i**) Anti-A3-LC antibody titers of A1-LC, A2-LC, or A3-LC antigens after the second immunization (**g**), after the third immunization (**h**), after the fourth immunization (**i**). The geometric mean titer (GMT) for each group was calculated using the average values. *n* = 5/group. Data are presented as mean ± SD. Statistical significance was determined by one-way ANOVA followed by Tukey’s post hoc test. * *p* < 0.05, ** *p* < 0.01, *** *p* < 0.001, **** *p* < 0.0001.

To further evaluate the humoral immune response, this study assessed the endpoint antibody titers (Figure 6). Consistent with the antibody level data, the average titers remained relatively low after the second immunization with low-dose antigens. When the plates were coated with A1-LC antigen, the A1-LC-His immunized group exhibited significantly higher antibody titers compared to the A2-LC-His and A3-LC-His groups (*p* < 0.05). Similarly, against the A2-LC coating antigen, the A2-LC-His group showed superior titers over the A1-LC-His and A3-LC-His groups (*p* < 0.05). When A3-LC was used as the coating antigen, the A3-LC-His immunized group generated significantly higher titers than the A1-LC-His and A2-LC-His groups (*p* < 0.05).

The antibody titers result further demonstrated that the fourth immunization elicited significantly higher antibody titers, indicating a progressively stronger immune response with successive immunizations. This finding is consistent with the antibody level data. Furthermore, a cross-protective effect was also evident. However, the overall antibody titers elicited by the A3-LC-His vaccine were lower than those induced by the A1-LC-His and A2-LC-His vaccines, which aligns with the observed outcomes in the protection assays.

### 2.5. Inhibition of BoNT/A LC Proteolytic Activity by Sera Antibodies

**Figure 7:**
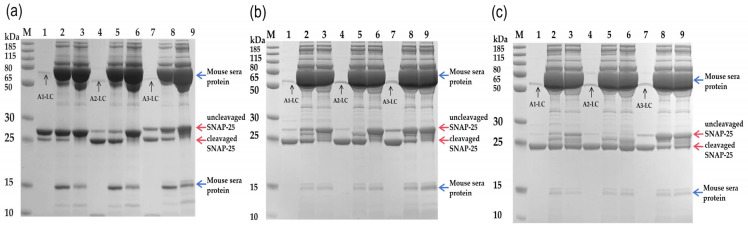
Cross-inhibition of BoNT/A light chain proteolytic activity by subtype-specific immune sera. The substrate SNAP-25 was fixed at 1.2 μM in all reactions. (**a**) Inhibition by anti-A1-LC sera. (**b**) Inhibition by anti-A2-LC sera. (**c**) Inhibition by anti-A3-LC sera. For each panel, the nine lanes are grouped and loaded as follows: Lanes 1–3 (A1-LC group): SNAP-25 + 9.4 nM (**a**) or 37.5 nM (**b**,**c**) A1-LC, supplemented with: lane 1, no sera; lane 2, 1 μL PBS-immunized sera (negative control); lane 3, 1 μL immune sera against the indicated LC. Lanes 4–6 (A2-LC group): SNAP-25 + 9.4 nM A2-LC, supplemented with: lane 4, no sera; lane 5, 1 μL PBS sera; lane 6, 1 μL immune sera against the indicated LC. Lanes 7–9 (A3-LC group): SNAP-25 + 100 nM (**a**) or 300 nM A3-LC (**b**, **c**), supplemented with: lane 7, no sera; lane 8, 1 μL PBS sera; lane 9, 1 μL immune sera against the indicated LC. Arrow indicators: blue arrows, mouse sera components; black arrows, light chains (LCs); red arrows, cleaved and uncleaved SNAP-25.

We have performed an assay to evaluate the ability of serum antibodies to neutralize light-chain enzymatic activity. In this assay, 1 μL of immune sera was pre-incubated with the respective LC before adding SNAP-25 substrate. As shown in Figure 7, anti-A1-LC and anti-A2-LC immune sera both effectively blocked most of the enzymatic activity of all three subtype LCs, demonstrating both neutralizing activity and broad cross-reactivity. Anti-A3-LC immune sera showed little to no blocking effect on the enzymatic activity of A1-LC or A2-LC, and only a partial blocking effect on its homologous A3-LC. In summary, these experiments demonstrate that anti-A1-LC and anti-A2-LC sera antibodies can effectively neutralize the enzymatic activity of three LCs, whereas anti-A3-LC sera antibodies fail to neutralize any of the three LCs, including their own enzymatic activity. This result demonstrates that while A3-LC is immunogenic, it neither elicits neutralizing antibodies nor provides protective immunity.

## 3. Discussion

The neurotoxic action of BoNT involves a four-step mechanism: binding to specific receptors on neuronal cells, followed by cellular internalization via endocytosis, translocation of the light chain into the cytosol, and finally, proteolytic cleavage of substrate proteins [24,25,26]. This process ultimately inhibits the release of acetylcholine, resulting in flaccid paralysis. It is LC, a zinc-dependent protease, cleaving SNARE proteins, that directly disrupts neuroexocytosis, inhibits acetylcholine release, and leads to flaccid paralysis [27,28,29]. Since the LC controls the catalytic properties and the duration of BoNTs action, variations in the LC will directly impact the therapeutic properties of BoNTs [7,30]. Consequently, the LC represents a critical domain for understanding BoNT pathogenicity and developing countermeasures.

This study focused on three BoNT/A subtypes (A1–A3). A1–A3 are among the earliest-identified subtypes and are of substantial research interest. Previous studies had shown that subtype A4 possesses very low biological activity [12], which placed it outside the immediate scope of this comparative analysis. Future studies are planned to extend this investigation to subtypes A4–A8 to provide a more comprehensive evaluation of their properties.

This work provides direct evidence that despite high sequence homology, the light chains (LCs) of BoNT/A1, A2, and A3 exhibit functional differences, as demonstrated by both in vitro substrate cleavage assays and in vivo immunoprotective efficacy in a mouse model. The core findings reveal that recombinant A1-LC-His and A2-LC-His possess superior proteolytic activity compared to A3-LC-His, as previously reported [12,29]. Moreover, immunization with A1-LC and A2-LC conferred robust protection against lethal challenge with BoNT/A1, A2, and A3 toxins, whereas A3-LC elicited markedly weaker protection. These results strongly suggest that intrinsic differences among the LCs substantially impact their potency as vaccine antigens.

The proteolytic activity profile determined in the experiment is consistent with the overall trends reported in prior research, although a little difference was observed. In the work of Whitemarsh et al. [12], the order of toxicity and cell-based activity for the native holotoxins was also A2 > A1 > A3, which is consistent with the functional difference that we report for the isolated LCs. Similarly, Henkel et al. [20] concluded that A3-LC was approximately 2-fold less active than LC/A1 and reported nearly equivalent activity between A2-LC and A1-LC, whereas our data show that A3-LC activity is nearly tenfold lower than that of A1-LC and A2-LC, while A2-LC exhibits approximately twice the activity of A1-LC. These discrepancies primarily stem from differences in the experimental systems employed. In contrast to studies utilizing native holotoxin [12], the present work employed recombinant light chains (LCs). This approach allowed us to bypass the heavy-chain-mediated steps of neuronal binding and intracellular translocation, thereby enabling a direct assessment of the intrinsic catalytic capacity of the LC domain toward its substrate, SNAP-25. When compared with the study by Henkel et al. [20], which also used recombinant protein expression, differences are noted. This divergence may be attributed to key differences in substrate and protein constructs: our study used full-length SNAP-25 (residues 1–206) and full-length LCs (1–448), whereas the aforementioned study employed a truncated SNAP-25 fragment (residues 141–206) and a truncated LC (residues 1–425). The truncated LC lacking a small C-terminal region may contribute to structural integrity and functional regulation. This may explain the variations observed in the proteolytic activity.

In the mouse protection model, both A1-LC and A2-LC elicited robust and broad protection against homologous and heterologous toxin challenges, including at doses as high as 100 LD_50_. In contrast, A3-LC failed to confer substantial protection even against its homologous toxin. Previous studies have documented notable differences in immunogenicity and protective efficacy among BoNT/A1-A3-Hc [19,31]. Our results extend these findings to the isolated LC domain, suggesting that the functional divergence among subtypes may be intrinsic to the core catalytic LC structure, which is different from the LC of recombinant native enzymatically inactivated BoNT/A [19,32].

In theory, the neutralizing epitopes of the light chain (LC) molecule are located in the catalytic active site, as well as in other regions of the LC. The neutralizing antibodies that target the enzymatic site can directly bind to and neutralize proteolytic activity. This effect is supported by the inhibitory activity of BoNT/A-LC to SNAP-25 in this study (Figure 7). In addition, the neutralizing epitopes in other LC regions may confer protection through alternative mechanisms—for example, by blocking intracellular delivery of the LC or by inducing conformational changes that allosterically impair its proteolytic activities [33,34].

The observed impairments in both the proteolytic activity and protective efficacy of A3-LC-His, particularly in contrast to A1-LC-His and A2-LC-His, raised a critical question: is this weakness an inherent property of the BoNT/A3-LC protein itself, or an artifact induced by its C-terminal fused His? To definitively rule out potential structural or functional interference from the His, this study designed and produced a parallel construct, A3-LC-TS, featuring a structurally distinct TS. The underlying rationale was that if His was responsible for the compromised efficacy, replacing it with an alternative tag should substantially rescue the protective phenotype. However, these results from both proteolytic activity assays and toxin challenge experiments demonstrated that A3-LC-TS performed comparably to A3-LC-His, with no observable significant improvement. This key evidence robustly indicates that His is not the primary cause of A3-LC’s diminished immunogenicity.

**Figure 8:**
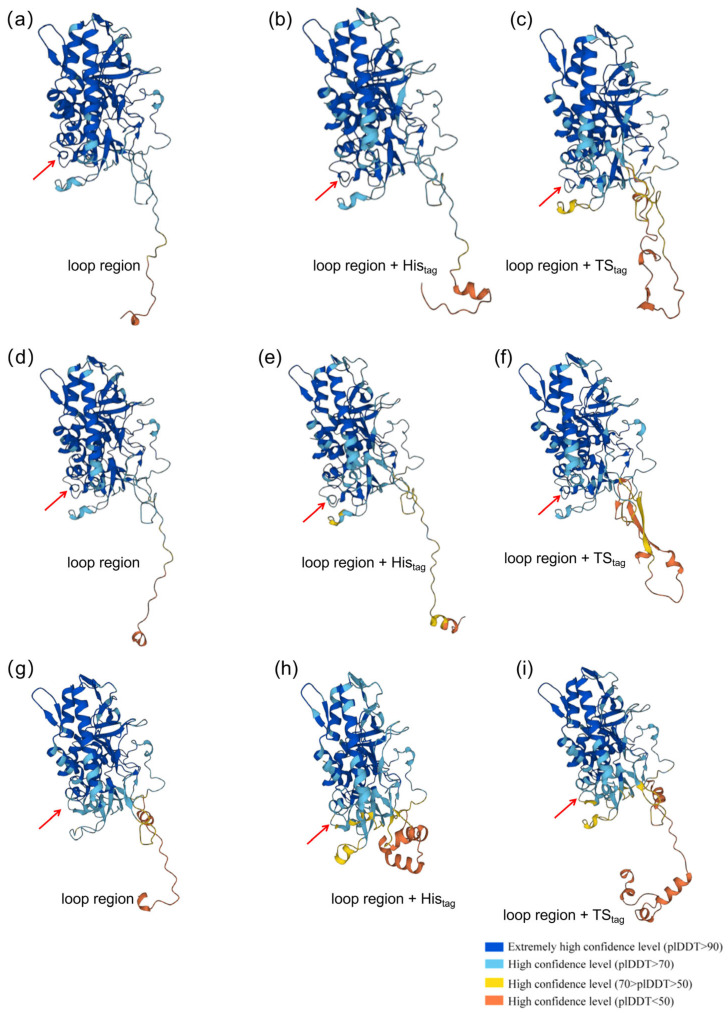
Protein structure prediction for the nine protein variants—untagged, His_tag_, and TS_tag_ forms of A1-LC, A2-LC, and A3-LC—was performed using the Paddle Helix biocomputing platform (https://paddlehelix.baidu.com) (27 October 2025). Red arrows indicate the four additional residues in A1-LC and A2-LC compared to A3-LC. (**a**) Protein structure prediction of A1-LC; (**b**) Protein structure prediction of A1-LC-His; (**c**) Protein structure prediction of A1-LC-TS; (**d**) Protein structure prediction of A2-LC; (**e**) Protein structure prediction of A2-LC-His; (**f**) Protein structure prediction of A2-LC-TS; (**g**) Protein structure prediction of A3-LC; (**h**) Protein structure prediction of A3-LC-His; (**i**) Protein structure prediction of A3-LC-TS.

To further corroborate this conclusion at the structural level, this study expanded our analysis to include systematic structural predictions for all nine protein variants: the untagged, His_tag_, and TS_tag_ versions of A1-LC, A2-LC, and A3-LC (Figure 8). The predicted structural models indicated that these two tags are situated within flexible regions of the protein molecules, distal from the catalytic active site and known neutralizing epitopes, and do not markedly alter the native folding of the core domain in any subtype. Therefore, this study concludes that the deficient efficacy of A3-LC is fundamentally rooted in its primary amino acid sequence. Structural analysis suggested a four-amino-acid deletion in A3-LC may underpin its impaired function. The four-amino-acid forms a loop in A1-LC and A2-LC, but not in A3-LC.

**Figure 9:**
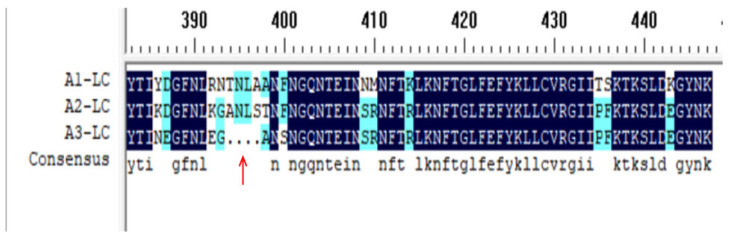
Sequence alignment of A1-LC, A2-LC and A3-LC. The red arrow indicates the four amino acids missing in A3-LC. A1-LC and A2-LC are derived from residues 1–448 of BoNT/A1 (*strain 62A*) and BoNT/A2 (*strain Kyoto*), respectively, whereas A3-LC originates from residues 1–444 of BoNT/A3 (*strain Banska Bystrica*).

Sequence alignment revealed a high homology of 95.09% between A1-LC and A2-LC. In contrast, the homology between A2-LC and A3-LC was 84.38%, and that between A1-LC and A3-LC was only 81.70% [20]. A notable structural distinction was observed: A1-LC and A2-LC consist of 448 amino acids, while A3-LC is four residues shorter, comprising only 444 amino acids (Figure 9). This study hypothesizes that this four-amino-acid deletion could be a critical factor contributing to the functional deficit of A3-LC. The deletion may induce subtle yet consequential alterations in local conformation or surface charge distribution, potentially impairing both catalytic efficiency and the presentation of immunodominant epitopes. For instance, although A1-LC, A2-LC, and A3-LC all cleave the substrate SNAP-25, they exhibit significant differences in catalytic activity and kinetics, which directly account for the functional divergence among the three subtypes. Ultimately, these structural and functional impairments may lead to the reduced enzymatic and protective performance of A3-LC compared to A1-LC and A2-LC. This hypothesis warrants further investigation in subsequent experiments.

Previous work from our lab [33,34,35,36,37] and another group [38] has shown that the L-HN domains of BoNT/A, B, E, and F serotypes can elicit a significant immunoprotective response in mice, thus supporting the significance of investigating the light chain domain. Building on this foundation, the present study systematically evaluated the functional characteristics of BoNT/A subtype LCs, thereby laying a solid foundation for future research into the enzymatic mechanisms and cross-protective immunity of LCs across various serotypes and their subtypes.

## 4. Conclusions

This study systematically compared the proteolytic activity and immunoprotective efficacy of the light chains from three BoNT/A subtypes. In vitro cleavage assays revealed that A2-LC exhibited the highest catalytic efficiency, followed by A1-LC, whereas A3-LC showed lower activity. In vivo protection experiments demonstrated that immunization with either A1-LC or A2-LC induced robust, broad-spectrum protection against challenges by all three homologous and heterologous toxin subtypes. In stark contrast, A3-LC elicited only minimal protection, which was confined to its homologous toxin. The diminished efficacy of A3-LC was confirmed to be an intrinsic property, not an artifact of the experimental design. Structural analysis indicates that a four-amino-acid deletion in A3-LC, compared to A1/A2-LC, likely underlies its impaired enzymatic function and protective immunogenicity.

In summary, this work clearly delineates systematic functional differences among the highly homologous BoNT/A light chains. These findings are crucial for advancing the mechanistic understanding of BoNTs and for developing next-generation countermeasures, including subtype-specific antitoxins and therapies. The validated success of A1 or A2-LC as a vaccine antigen highlights its potential as a key component in future subunit-based vaccines, potentially offering broader or more potent protection.

## 5. Materials and Methods

### 5.1. Gene Synthesis

Gene synthesis of the A1-LC, A2-LC, and A3-LC DNA fragments according to their amino acid sequences was performed by BGI Genomics Co., Ltd. (Shenzhen, China). These DNA fragments were subsequently cloned into the pTIG-Trx-His and pTIG-Trx-TS vectors via double digestion with restriction enzymes EcoR I and Xho I. This procedure yielded four recombinant plasmids: pTIG-Trx-A1-LC-His, pTIG-Trx-A2-LC-His, pTIG-Trx-A3-LC-His, and pTIG-Trx-A3-LC-TS. The relevant information for the A1-LC, A2-LC, and A3-LC is summarized in Table 3.

### 5.2. Protein Expression

The four recombinant plasmids were individually transformed into *E. coli* BL21 (DE3) competent cells (TransGen Biotech, Beijing, China). Positive clones were selected and inoculated into 6 mL of 2 × YT medium supplemented with 100 μg/mL ampicillin, followed by incubation at 37 °C with shaking at 220 rpm. When the OD_600_ reached 0.6–1.0, the cultures were diluted 1:100 into 400 mL of fresh 2 × YT medium containing the same antibiotics and grown under identical conditions until the OD_600_ again reached 0.6–1.0. Protein expression was induced by adding isopropyl β-D-thiogalactoside (IPTG; Tiangen Biotech, Beijing, China) to a final concentration of 0.2 mM, and the cultures were further incubated overnight at 16 °C with shaking. Bacterial cells were harvested by centrifugation at 8000 rpm for 5 min. The cell pellets were resuspended in binding buffer and lysed via sonication on ice. The lysates were then centrifuged at 8000 rpm for 10 min at 4 °C to collect the soluble fraction (supernatant), which was subsequently filtered through a 0.22 μm membrane.

### 5.3. Protein Purification

Purification of His_tag_ proteins was performed using an ÄKTA go chromatography system. A HisTrap HP column (Zhongyuan Heju Biotechnology Co., Ltd., Beijing, China) was first washed with ultrapure water and then equilibrated with binding buffer (20 mM Tris-HCl, 500 mM NaCl, 20 mM imidazole, pH 8.0). The filtered supernatant was then loaded onto the column. After washing with the binding buffer containing 1 M NaCl to remove non-specifically absorbed impurities, the bound proteins were eluted using a linear gradient with elution buffer (20 mM Tris-HCl, 500 mM NaCl, 500 mM imidazole, pH 8.0) to obtain target proteins and remove weakly bound impurities. The HisTrap HP column was cleared using 1 M NaOH. The eluted fractions were collected and dialyzed into PBS (pH 7.4). The purity and identity of the proteins were analyzed by 10% SDS-PAGE, and the purified proteins were stored at −20 °C.

For Twin-Strep-tagged (TS_tag_) proteins, purification was carried out using a StrepTrap XT column. The binding buffer consisted of 100 mM Tris-HCl, 150 mM NaCl, 1 mM EDTA (pH 8.0), and the elution buffer was 100 mM Tris-HCl, 150 mM NaCl, 1 mM EDTA, 50 mM biotin (pH 8.0). The remaining steps were consistent with the procedure described for His_tag_ protein purification.

### 5.4. Western Blot Analysis

Purified recombinant proteins (A1-LC, A2-LC and A3-LC), with a loading amount of 0.3 μg per lane, were separated by 15% SDS-PAGE and subsequently transferred onto a PVDF membrane using a semi-dry transfer system. The membrane was blocked with 5% skim milk in TBST (Tris-buffered saline with 0.1% Tween-20) for 2 h at room temperature. It was then incubated with 7 mL of a 1:500 dilution of anti-A1-LC (anti-BoNT/A horse sera), A2-LC, or A3-LC mouse sera antibodies in TBST for 1.5 h at room temperature. Following incubation, the membrane was washed six times for 5 min each with TBST. Subsequently, it was incubated with 7 mL of a 1:5000 dilution of the corresponding horseradish peroxidase (HRP)-conjugated secondary antibody (goat anti-horse IgG for A1-LC blots or goat anti-mouse IgG for A2/A3-LC blots; Zhongshan Jinqiao, Beijing, China) in TBST for 30 min at room temperature. After another six washes with TBST, the blot was developed using an ECL chemiluminescence detection kit (Thermo Fisher Scientific, Waltham, MA, USA) and visualized by scanning.

### 5.5. Proteolytic Activity

The substrate protein SNAP-25 was expressed in *E. coli* and purified via His affinity chromatography, with its purity verified by SDS-PAGE [35]. In a 50 μL reaction system, 2.4 μM SNAP-25 substrate was incubated with serially diluted recombinant protein (molar ratios ranging from 2:1 to 1:2048) in cleavage buffer (50 mM HEPES, pH 7.5, 10 mM NaCl, 0.1% Tween-20, 5 mM DTT) at 37 °C for 30 min. The reaction was terminated by adding 12.5 μL of 5× SDS-PAGE, reducing the loading buffer and heating at 95 °C for 5 min. A 25 μL aliquot of the mixture was then separated by 15% SDS-PAGE, and the cleavage bands were quantified using grayscale analysis with ImageJ 1.54g software (NIH, Bethesda, MD, USA) to evaluate proteolytic efficiency.

### 5.6. Mouse Immunization and Protection Assay

Female BALB/c mice (6–8 weeks old, SPF grade) purchased from SPF (Beijing) Biotechnology Co., Ltd. were divided into experimental groups and were raised at the Laboratory animal center, Academy of Military Medical Sciences, Beijing, China, under clean-grade conditions. All animal experiments and related operations were in accordance with the ethical standards for laboratory animals and were reviewed and approved by the Academy of Military Medical Sciences Experimental Animals committee (LACU-DWZX-2024-034) and Beijing Institute of Biotechnology Experimental Animals committee (IACUC-SWGCYJS-2025-048). Five mice per group were used in all experiments of this study according to the principle of reduction. These mice were immunized with 1 μg or 10 μg antigen three or four times with two weeks interval. A control group injected with PBS only was included. Blood samples were collected from all mice prior to each immunization and challenge for subsequent analysis of antibody levels and titers. The number of surviving mice and symptoms of botulism (Muscle paralysis, expiratory dyspnea and general spasms, etc.) were recorded every day for four days.

The 50% lethal dose (LD_50_) for each botulinum neurotoxin subtype used in this study had been determined in the previous article [35,39]. The LD_50_ was determined as follows: The neurotoxicity of each toxin in 18~20 g mice (four mice per concentration level) was determined using an LD_50_ assay. These toxins were diluted with sterile normal saline. Each mouse was intraperitoneally injected with 500 μL of serially diluted toxins. The number of surviving mice and symptoms of botulism were recorded every day for four days. The minimum amount of toxin that killed 50% of the mice within four days was defined as the LD_50_, expressed as LD_50_/mL or LD_50_/mg, and the toxicity of BoNT/A1-A3 is 4.26 × 10^6^ LD_50_/mL, 3.85 × 10^4^ LD_50_/mL, 9.59 × 10^4^ LD_50_/mL, respectively. Mice were challenged via intraperitoneal injection with 500 μL of toxin solution at three weeks after the final immunization. The self-protection effect was assessed first with homologous toxin, followed by a cross-challenge to evaluate protection against other toxin serotypes. For the protection experiments, 500 μL of toxin solutions containing 10, 100, or 1000 LD_50_ of the respective toxin were prepared directly according to the corresponding doses.

### 5.7. Enzyme-Linked Immunosorbent Assay (ELISA)

Serum levels and antibody titers of specific antibodies against A1-LC, A2-LC, and A3-LC antigens were determined by ELISA. Briefly, 96-well plates were coated with 100 μL/well of A1-LC, A2-LC, or A3-LC antigen (2 μg/mL) and incubated overnight at 4 °C. After discarding the coating solution, plates were blocked with 200 μL/well of 2% bovine serum albumin (BSA) at 37 °C for 2 h, followed by six washes with PBST (PBS containing 0.1% Tween-20). For antibody level determination, 100 μL of a 1:100 dilution of mouse serum was added to each well. For titer determination, serum samples were serially diluted fourfold starting from 1:100. Plates were incubated at 37 °C for 1.5 h, washed, and then incubated with 100 μL/well of a 1:5000 dilution of HRP-conjugated goat anti-mouse IgG secondary antibody (Zhongshan Jinqiao, Beijing, China) at 37 °C for 30 min. After final washing, 50 μL of chromogenic substrate solution (47% 0.2 M Na_2_HPO_4_, 43% 0.1 M citric acid, 9% 10× OPD, 1% 30% H_2_O_2_) was added and incubated in the dark at room temperature for 8 min. The reaction was stopped by adding 50 μL of 2 M H_2_SO_4_, and the absorbance was immediately measured at 492 nm using a microplate reader. Serum samples of each mouse in all immunization groups were measured separately, and the serum antibody levels and titers for each group were calculated using the average values.

### 5.8. Inhibitory Activity of BoNT/A LC to SNAP-25 In Vitro Tests

An in vitro assay was performed to assess the inhibitory activity of serum antibodies against the proteolytic function of BoNT/A light chains (LCs) on the substrate SNAP-25. All reactions were conducted in a 50 mM PBS buffer with a total volume of 50 µL. First, 1 µL of immune sera was pre-incubated with 10 µL of the respective BoNT/A LC (9.4 nM or 37.5 nM A1-LC, 9.4 nM A2-LC, 100 nM or 300 nM A3-LC) at 37 °C for 1 h. Subsequently, 1.2 µM of SNAP-25 substrate was added, and the mixture was incubated for an additional 1 h at 37 °C. Reactions were terminated by adding SDS-PAGE loading buffer, and the products were analyzed by electrophoresis on a 15% polyacrylamide gel.

### 5.9. Statistical Analysis

All data were analyzed using GraphPad Prism (Version 8.0.2, GraphPad Inc., San Diego, CA, 532 USA) and SPSS software (Version 19.0, IBM Corp., Armonk, NY, USA). The quantitative data of experimental results is expressed as mean ± standard deviation (SD). Differences in antibody immune responses between groups were assessed using one-way analysis of variance followed by Tukey’s honest significant difference (HSD) post hoc test for multiple comparisons. Fisher’s exact test was used to determine the statistical difference in survival between the treatment groups. In all analyses, probability (*p*) values below 0.05 were defined as statistically significant.

## Figures and Tables

**Table 1:** Protective potency after three immunizations with A1-LC, A2-LC and A3-LC antigens in mice.

Vaccine (10 μg) ^a^	Number Alive ^b^		
	10 LD_50_ A1	10 LD_50_ A2	10 LD_50_ A3
A1-LC	5/5 **	4/5 *	5/5 **
A2-LC	5/5 **	5/5 **	5/5 **
A3-LC-His	ND	ND	0/5
A3-LC-TS	ND	ND	0/5
PBS	0/5	0/5	0/5

ND means not determined. ^a^ Mice (*n* = 5/group) were immunized three times with 1 μg and 10 μg of A1-LC, A2-LC and A3-LC antigens, respectively. PBS was used as a negative control. ^b^ Mice (*n* = 5/group) were challenged with toxins after three immunizations. The number of survivors is shown. * *p* = 0.0476 < 0.05, ** *p* = 0.0079 < 0.01, compared with negative control (PBS) or A3-LC group. Statistical comparisons of survival rates were performed using Fisher’s exact test.

**Table 2:** Protective potency after four immunizations with A1-LC, A2-LC, and A3-LC antigens in mice.

Vaccine (Dose) ^a^	Number Alive ^b^			
	10^2^ LD_50_A1	10^2^ LD_50_A2	10^2^ LD_50_A3	10^3^ LD_50_A1
A1-LC (1 μg)	5/5 **	3/5	2/5	ND
A1-LC (10 μg)	5/5 **	4/5 *	4/5 *	5/5
A2-LC (1 μg)	4/5 *	4/5 *	4/5 *	ND
A2-LC (10 μg)	5/5 **	5/5 **	5/5 **	5/5
A3-LC (1 μg)	0/5	0/5	1/5	ND
A3-LC (10 μg)	0/5	0/5	4/5	ND
A3-LC-TS (1 μg)	ND	ND	0/5	ND
A3-LC-TS (10 μg)	ND	ND	0/5	ND

ND means not determined. ^a^ Mice (*n* = 5/group) were immunized four times with 1 μg and 10 μg of A1-LC, A2-LC and A3-LC antigens, respectively. ^b^ Mice (*n* = 5/group) were challenged with toxins after four immunizations. The number of survivors is shown. * *p* = 0.0476 < 0.05, ** *p* = 0.0079 < 0.01, compared with A3-LC group. Statistical comparisons of survival rates were performed using Fisher’s exact test.

**Table 3:** Basic information on recombinant LC functional fragments of BoNT/A.

Subtype	Amino Acid Sequence	Base Pairs (bp)	Protein Molecular Weight
A1-LC	1–448	1344	50 kDa
A2-LC	1–448	1344	50 kDa
A3-LC	1–444	1332	50 kDa

## Data Availability

The original contributions presented in this study are included in the article. Further inquiries can be directed to the corresponding authors.
